# Case Report of Multiembolic Cerebrovascular Event Associated with Ramp Study Echocardiogram

**DOI:** 10.1155/2017/9072523

**Published:** 2017-09-28

**Authors:** Brian C. Butera, Luanda P. Grazette, Tracy Lawrence, Michael E. Bowdish, Andrew J. Yoon

**Affiliations:** ^1^Department of Internal Medicine, Division of Cardiovascular Medicine, University of Southern California, Los Angeles, CA, USA; ^2^Department of Surgery, Division of Cardiothoracic Surgery, University of Southern California, Los Angeles, CA, USA

## Abstract

The incidence of ramp test echocardiogram-associated embolic events in the setting of therapeutic anticoagulation is likely rare and has not been reported. We present such a case in a patient with a HeartMate II left ventricular assist device (LVAD) whose serial head computed tomography images, deteriorating clinical course, and the multiembolic nature of the event suggest causality. If the pretest probability of pump thrombosis in an individual LVAD patient is sufficiently high, the potential risks of performing a ramp study echocardiogram may not be warranted, even in the setting of adequate anticoagulation.

## 1. Introduction

The ramp test echocardiogram is a noninvasive test that can diagnose pump thrombosis by comparing expected versus actual left ventricular end-diastolic volume (LVEDD) at different LVAD speeds [1, 2]. It is one of the few research validated imaging methods available for diagnosing LVAD pump thrombosis. While current guidelines warn against performing ramp tests in patients with subtherapeutic anticoagulation given the risk of internal thrombus liberation [3], this test is not traditionally considered a risk factor for thromboembolization in the setting of therapeutic anticoagulation. We present, to our knowledge, the first case of multiembolic cerebrovascular event following a ramp test echocardiogram in a patient with appropriate therapeutic anticoagulation.

## 2. Case Presentation

A 64-year-old Caucasian male with ischemic cardiomyopathy and 2 prior coronary bypass-grafting operations underwent implantation with a HeartMate II LVAD as destination therapy. He had an asymptomatic internal pump thrombus diagnosed 35 months after implantation, when outpatient lactate dehydrogenase (LDH) levels abruptly increased from 400 h/L to 1027 u/L in the setting of a subtherapeutic International Normalized Ratio (INR) of 1.8. LDH levels rapidly returned to normal after a short treatment course of intravenous (IV) Heparin. The patient continued aspirin and warfarin, and his INR was therapeutic on subsequent lab tests (target INR 2.2–2.7).

**Figure 1:**
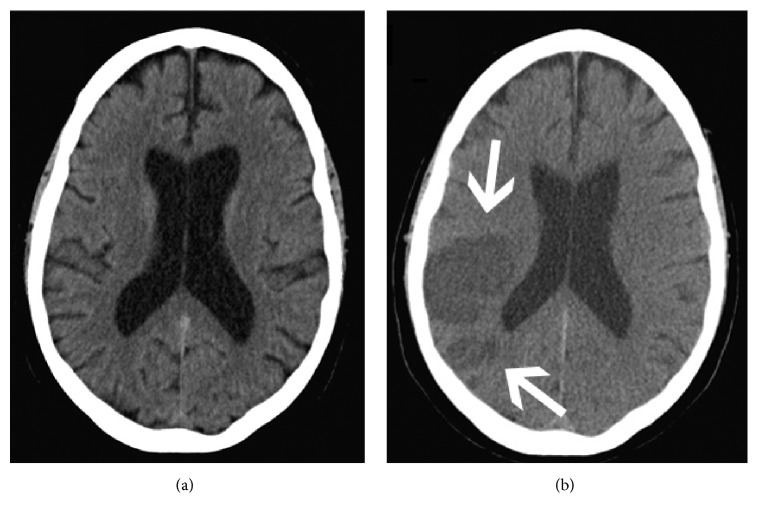
(a) Head CT at time of admission showing no acute findings. (b) Subsequent head CT showing acute multiembolic stroke (arrows).

Five months later, the patient presented in extremis to an outside hospital in respiratory distress and fluid overload and was intubated and eventually transferred to our institution for a higher level of care. Sputum was positive for Influenza A. Tamiflu and IV antibiotics were started; subsequent blood cultures were negative. Initial INR was 2.9, LDH was 1429 units/L, and haptoglobin was < 10 mg/dL. IV diuretics were started. Initial head computed tomography (CT) scan showed no new or acute processes (Figure 1(a)). Interrogation of the patient's implantable cardioverter defibrillator revealed no evidence for significant arrhythmias. LVAD interrogation recorded a transient power spike to 9 Watts in the days prior to admission (baseline power = 5.5 Watts).

**Figure 2:**
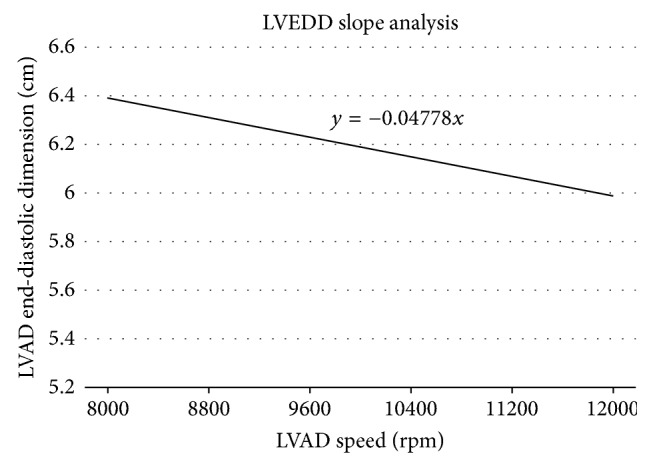
Ramp study analysis showing LVEDD slope of −0.05. An LVEDD slope ≥ −0.16 is consistent with flow obstruction due to pump thrombosis. LVEDD = left ventricular end-diastolic dimension. RPM = revolutions per minute.

Given the history and findings, a ramp test echo was performed to confirm the diagnosis of LVAD thrombus. Preceding ramp echo testing, standard transthoracic echocardiography was used to verify the absence of intracardiac or aortic root thrombus. From a baseline LVAD speed of 9000 rpm, echocardiogram images were then obtained in stepwise increments from 8600 rpm to 12000 rpm (Figure 2). The aortic valve did not open at any speed. The left ventricular end-diastolic dimension (LVEDD) slope was −0.05, and the pulsatility index (PI) slope was −0.18, consistent with an internal pump thrombus [1]. Despite the therapeutic INR, the decision was made to start an IV Heparin drip. Integrilin was not initiated due to a positive test for fecal occult blood and a slowly downtrending hemoglobin level. Pump exchange was not performed due to the hostile mediastinum.

The patient was initially stable after the ramp test, but his clinical status rapidly deteriorated over the subsequent 3 days. LDH levels remained highly elevated. Escalating doses of IV milrinone and epinephrine were started for escalating multiorgan failure in the setting of cardiogenic and septic shock. A repeat head CT scan revealed multiple acute embolic infarcts in the bilateral posterior temporal lobes, left occipital lobe, and posterior right middle cerebral artery involving the parietal and temporal lobes (Figure 1(b)). Serial blood cultures remained negative and repeated echocardiogram imaging did not suggest endocarditis. Given the poor prognosis in conjunction with the new CT findings, the family agreed to withdraw medical care and declined autopsy.

## 3. Discussion

Of 45 total ramp study echocardiograms performed at our institution, this case is noteworthy for the timing of the ramp study in relation to the multiembolic stroke. It is difficult to directly attribute stroke causality to a ramp test unless the event is immediately, catastrophically apparent. This patient was intubated and sedated, minimizing significant clinical examination findings. The proximity of consecutive head CT images in relation to the ramp study in our case, as well as the multiembolic nature of the event, is suggestive of (briefly delayed) causation. In addition, our patient was actively infected with Influenza A, and infection has been identified as a risk factor for neurologic events in the LVAD population [4–6]. The patient's anticoagulation was also therapeutic at time of presentation, but as is often the case, his anticoagulation status in the medium term cannot be certain. Although peripherally located thrombus embolization from the carotid bulb has been described [7], the multivascular distribution of the cerebral infarcts suggests a more central source.

At present, there are few contraindications to ramp echo, allowing for proactive and often liberal employment of this testing modality. While baseline, preramp imaging is important for device screening, clinical suspicion for thrombus should guide the decision for further testing as speed manipulation creates transient variation in an otherwise continuous flow state, leading to fluctuations in shear strain force [8]. We postulate that this may amplify hemorheological factors such as heat production, platelet activation, and turbulence, which are already recognized as contributory to thrombus formation in LVAD patients [9]. Variation in shear strain force may also lead to thrombus instability and account for potential liberation from the site of the device blood interface. Ultimately, when the pretest probability of pump thrombosis is abundantly high, or if the risk threshold of the ramp study is sufficiently altered by the presence of significant comorbidities, the potential hazards of performing a ramp study echocardiogram may outweigh the diagnostic benefits, even in the setting of chronically therapeutic anticoagulation. A multi-institutional registry quantifying the incidence of embolic strokes occurring in temporal relation to ramp study echocardiograms, with INR correlation, could provide illumination.
